# Remote Monitoring of Cryosurgery Response Using a Smartphone App: Prospective Study

**DOI:** 10.2196/63467

**Published:** 2026-03-18

**Authors:** Vanessa R Weir, Emily A Cowen, Trina Salvador, Mary D Sun, Lilly Gu, Maura C Gillis, Nicholas R Kurtansky, Veronica Rotemberg, Allan C Halpern

**Affiliations:** 1 Dermatology Service, Memorial Sloan Kettering Cancer Center, 530 E 74th St, New York, NY, 10021, United States, 1 212-639-2000

**Keywords:** cryosurgery, cryotherapy, keratoses, tele-dermatology, remote monitoring, local skin response, lesions, patient care, mobile imaging, erythema, skin response

## Abstract

**Background:**

Cryosurgery is an effective treatment for benign lesions, although current unstandardized approaches may result in inadequate responses and unwanted adverse reactions. Monitoring treatment characteristics, lesion responses, and patient-reported outcomes using patient-derived mobile imaging may facilitate longitudinal treatment assessment.

**Objective:**

This study aimed to determine the reliability of metrics for assessing the response to cryosurgery in patients with actinic and seborrheic keratoses using remote photographic monitoring.

**Methods:**

Patients who were recommended cryosurgery by their physician for treating seborrheic and/or actinic keratoses (22 patients with 31 lesions) were enrolled. After treatment, participants took “overview” and “close up” photos of their lesion(s) and rated appearance, pain, and degree bothered on a custom smartphone app at eight posttreatment time points (days 0, 3, 7, 10, 14, 30, 60, and 90). After study completion, independent raters scored the images for local skin response (eg, erythema, scaling, crust, swelling, vesiculation, and erosion), cosmetic outcome (eg, hyperpigmentation, hypopigmentation, scarring, and atrophy), and lesion resolution.

**Results:**

The local skin response peaked 3 days after cryosurgery, with 26% (7/27) of patients reporting pain. There was substantial agreement between raters for lesion resolution (κ=0.71, 95% CI 0.62‐0.79), erythema (κ**=**0.66, 95% CI 0.57-0.74), and the local skin response index (κ**=**0.69, 95% CI 0.61-0.77) as measured using the quadratic-weighted Cohen κ. Overall, 77% (151/195) of submitted photos were good quality, and most image-derived metrics showed higher agreement in good-quality photos (8/14, 57% metrics had moderate-substantial κ) compared to poor-quality photos (4/14, 29% metrics had moderate-substantial κ). The peak local skin response had a moderate positive association with the lesion response at 90 days (Spearman ρ=0.556, *P*=.01).

**Conclusions:**

This study demonstrates the utility of patient self-imaging for longitudinal assessment of the response to cryosurgery.

## Introduction

Nonsurgical therapies remain the mainstay for many symptomatic benign or precancerous lesions, including actinic or seborrheic keratoses. Cryosurgery is an effective treatment for isolated keratoses and remains the most commonly used destructive modality [1]. However, current unstandardized approaches to freezing techniques may result in inadequate responses and unwanted adverse reactions [2-4]. This highlights the need for more systematic approaches that can optimize treatment outcomes while incorporating patient preferences.

Store-and-forward mobile apps are increasingly used for therapeutic evaluation and research photo-documentation in dermatology [5-7]. This technology offers benefits by reducing geographical limitations and time constraints that make longitudinal research difficult. Beyond facilitating remote lesion monitoring, they can be utilized to document cutaneous events in response to therapeutics while decreasing barriers to follow-up. Our study aimed to evaluate the agreement of image-derived grading of lesion responses and to investigate their correlation with patient-reported adverse reactions in those with actinic and seborrheic keratoses treated with cryosurgery utilizing patient-submitted images.

## Methods

### Study Design

This was a prospective, single-center, observational study undertaken at the Memorial Sloan Kettering dermatology clinic in New York City from October 2021 to June 2023. Patients were included if they were at least 18 years of age with at least one seborrheic or actinic keratosis undergoing destructive treatment and who could either take a photo of their lesion themselves or have a partner do so. Exclusion criteria included not having access to an iPhone or the inability of the patient or their partner to photograph the lesion. Prior to the study, patient eligibility was assessed.

### Ethical Considerations

This study was reviewed and approved by the Memorial Sloan Kettering Cancer Center’s Institutional Review Board (Protocol #21‐019). All participants provided written informed consent for participation and publication of their case details, and the research was conducted in accordance with principles embodied in the Declaration of Helsinki and in accordance with local requirements. Analytic data and images submitted via the mobile app were linked to deidentified study identifiers only. Participants received no financial compensation for participation in the study.

### Data Collection

Patients were trained to use a smartphone-based self-imaging app (Canfield Capture Mobile App; Canfield Scientific, Inc). Before cryosurgery, participants took baseline overview and close-up photographs of up to three lesions in clinic using the app. After receiving provider-administered cryosurgery using variable techniques, patients were asked to remotely continue photographing lesions and complete a symptom questionnaire (rating pain, degree bothered, and cosmesis) at eight posttreatment time points (days 0, 3, 7, 10, 14, 30, 60, and 90) using the app. A standardized imaging protocol emphasizing consistent lighting, positioning, and focus was suggested. Full instructions, photo quality checklists, and questionnaire items given to the patients are available in Multimedia Appendix 1.

### Independently Rated Measurements

Paired pre- and posttreatment images were independently reviewed by two board-certified dermatologists using a structured scoring rubric. Reviewers assessed lesion resolution, local skin response (LSR; including erythema, crusting, swelling, vesiculation/pustulation, erosion/ulceration, and flaking/scaling), and photo quality (good vs poor) across the eight time points. Each image pair was assigned both individual scores (0-4) and a composite LSR index (range 0-24). The lesion response was classified as a binary outcome (complete vs incomplete) and using a four-point scale, which were both used to score the response. Ratings were completed using a standardized interface with anonymized, time-randomized images to reduce bias. The full scoring criteria, interface set up, and workflow can be found in Multimedia Appendix 2.

### Statistical Analysis

The primary objective was to assess whether lesion resolution can be reliably evaluated through patient-captured photographs. The quadratic-weighted Cohen κ was used to measure the interrater agreement of lesion resolution (incomplete vs complete), as well as other visually determined posttreatment cutaneous gradings across all time points. The Spearman rank correlation was used to evaluate the relationships between the physician-rated skin responses and the patient-reported adverse reactions. A principal component analysis was conducted to capture significant variance and patterns in the data collected for peak response values and lesion outcomes at day 90. All statistical analysis was performed with R software (version 4.3.1; R Foundation for Statistical Computing) using the following packages: *dplyr*, *tidyverse*, *psych*, *ggplot2*, *stats*, and *table1*.

## Results

### Study Population

A summary of patient characteristics can be found in Table 1. We enrolled 22 patients with 31 total lesions (18 seborrheic keratoses and 13 actinic keratoses). Patients had Fitzpatrick skin types II-IV. The cryosurgery apertures used included A, B, C, 20 gauge, 22 gauge, and angiocath. Lesions were treated with liquid nitrogen using a mean distance of 1.63 (range 1.00-3.00) cm, 1 or 2 cycles, with an average spray time of 9.84 (SD 5.46) seconds. At 90 days, the participation rate was 68% (21/31 lesions).

**Table 1.** Demographic characteristics.

	Seborrheic keratosis (n=18), n (%)	Actinic keratosis (n=13), n (%)	Overall (N=31), n (%)
Age range (years)			
50‐64	5 (27.8)	3 (23.1)	8 (25.8)
65‐79	13 (72.2)	8 (61.5)	21 (67.7)
≥80	0 (0)	2 (15.4)	2 (6.5)
Skin type			
II	12 (66.7)	12 (92.3)	24 (77.4)
III	5 (27.8)	1 (7.7)	6 (19.4)
IV	1 (5.6)	0 (0)	1 (3.2)
Site			
Head/neck	8 (44.4)	4 (30.8)	12 (38.7)
Anterior torso	1 (5.6)	1 (7.7)	2 (6.5)
Posterior torso	2 (11.1)	0 (0)	2 (6.5)
Lateral torso	4 (22.2)	0 (0)	4 (12.9)
Upper extremity	0 (0)	5 (38.5)	5 (16.1)
Lower extremity	3 (16.7)	3 (23.1)	6 (19.4)
Lesions available for analysis^a^			
Day 0	16 (88.9)	12 (92.3)	28 (90.3)
Day 3	17 (94.4)	10 (76.9)	27 (87.1)
Day 7	17 (94.4)	9 (69.2)	26 (83.9)
Day 10	17 (94.4)	10 (76.9)	27 (87.1)
Day 14	16 (88.9)	8 (61.5)	24 (77.4)
Day 30	16 (88.9)	8 (61.5)	24 (77.4)
Day 60	17 (94.4)	5 (38.5)	22 (71.0)
Day 90	15 (83.3)	6 (46.2)	21 (67.7)

a Completion rate defined as the proportion of treated lesions with evaluable image submissions available at each posttreatment time point, relative to the total number of treated lesions at baseline.

### Cryosurgery Efficacy and Tolerability

**Figure 1.**
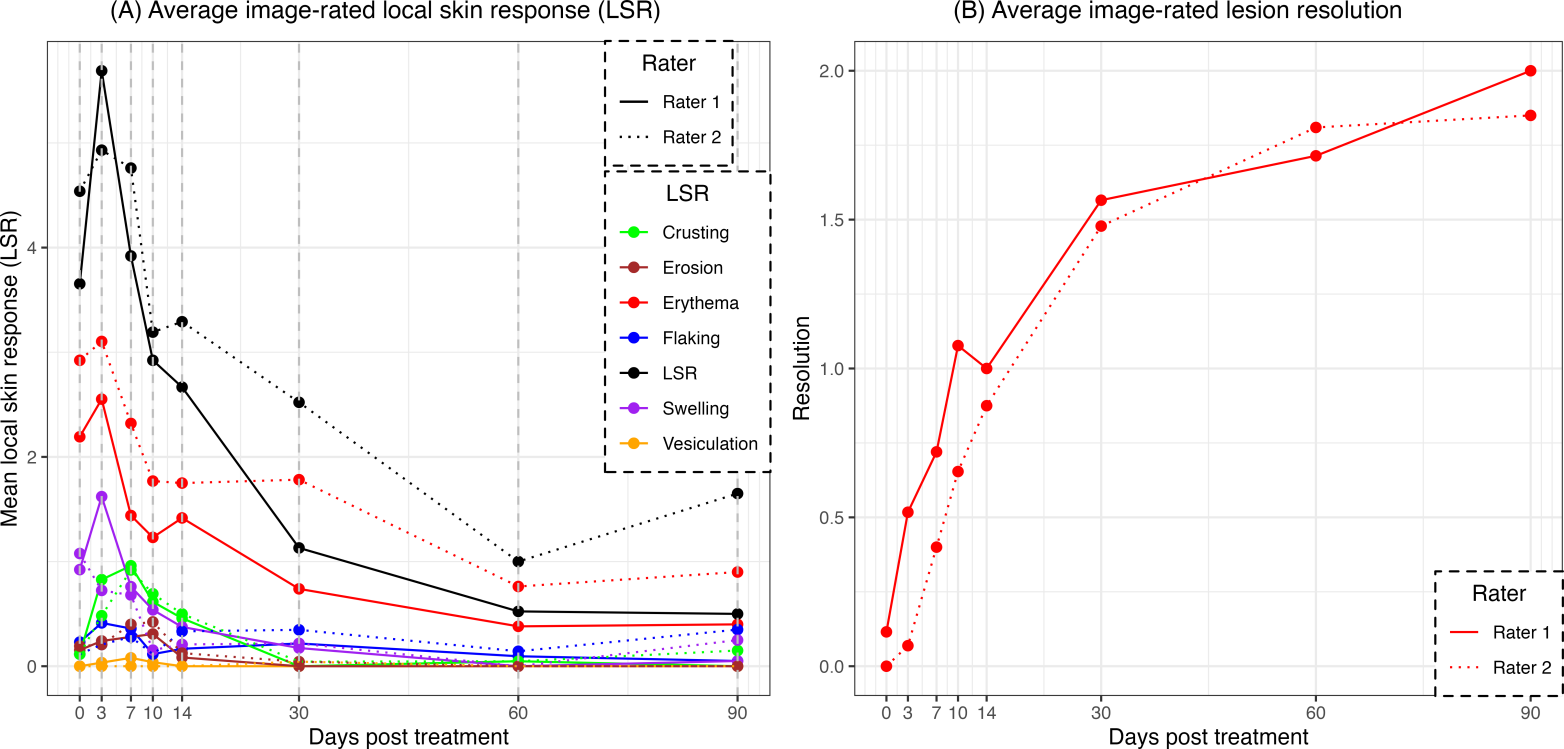
Average image-rated local skin response (LSR) following cryosurgery treatment. (A) The mean image-rated LSR for rater 1 and rater 2 following cryosurgery. The individual LSR metrics were rated on a scale of 0 (none or at baseline) to 4. The LSR is a composite of the scores for crusting, erosion, erythema, scaling or flaking, swelling, and vesiculation (0‐24). The LSR was the highest between days 0 and 7 after treatment, peaking on day 3, and largely resolved by day 60. Erythema presented as the predominant symptom, while vesiculation was the least common. (B) The mean image-rated lesion response for rater 1 and rater 2 following cryosurgery. The lesions were rated from 0 (no change) to 3 (complete resolution). At 90 days, 58% (11/19) of the lesions were considered resolved by at least one rater.

The frequency of image-rated metrics across all time points is listed in Multimedia Appendix 3. Erythema, flaking, scaling, crusting, and swelling were observed in >50% of lesions, with erythema (27/29, 93%) and swelling (24/29, 83%) being the most commonly observed effects. Vesiculation, atrophy, and scarring were observed in ≤10% of lesion responses. Figure 1A shows the time course of the mean LSR for rater 1 and rater 2. The LSR peaked at day 3, with erythema having the highest mean (mean 2.58, SD 1.3 for rater 1, and mean 3.10, SD 1.5 for rater 2) and vesiculation having the lowest mean (mean 0.04, SD 0.2 for rater 1, and mean 0.00, SD 0.0 for rater 2). Figure 1B shows the average lesion response over time. At 90 days, 32% (6/19) of the lesions were considered resolved by both raters and 58% (11/19) of the lesions were considered resolved by at least one rater.

Multimedia Appendix 4 shows the patient-rated adverse reactions over time. At 3 days, 26% (7/27) of the patients reported pain, 19% (5/27) reported being bothered by adverse reactions, and 37% (10/27) reported cosmetic outcomes as “poor” or “fair.” At 90 days, 71% (15/21) of the patients reported no pain, 76% (16/21) reported very good or excellent cosmetic outcomes, and 100% (21/21) reported they were not bothered by adverse reactions.

### Reliability of Image-Rated Metrics

Table 2 shows the interrater agreement for image-derived metrics of the cryosurgery response. Overall, there was substantial agreement for lesion resolution using a four-point scale (κ**=**0.71), composite LSR (κ**=**0.69), and erythema (κ**=**0.66). Vesiculation, hyperpigmentation, hypopigmentation, and atrophy had negligible agreement.

Table 3 shows the interrater agreement by photo quality. Good-quality photos (n=151) consisted of photo sets where the quality was graded as “good” by both raters. Poor-quality photos (n=44) consisted of sets where at least one person graded the quality as “poor.” Lesion resolution (scored as completed or incomplete) was more reliable for good-quality photos compared to poor-quality photos (κ**=**0.64 vs κ**=**0.14). Although several image-rated response metrics had higher agreement in good-quality photos (including the LSR index, erythema, erosion, scaling, and swelling), crusting, flaking, and hyperpigmentation had similar or slightly higher agreement in poor-quality photos.

**Table 2.** Interrater agreement for image-derived metrics of cryosurgery response (n=195).

Image derived metric	κ value^a^	95% CI
Four point scale		
Lesion resolution^b^	0.71	0.62 to 0.79
Complete versus incomplete		
Lesion Resolution	0.56	0.41 to 0.72
Local skin response metrics		
Erythema	0.66	0.57 to 0.74
Crusting	0.52	0.35 to 0.69
Local skin response composite	0.69	0.61 to 0.77
Erosion	0.47	0.20 to 0.73
Scaling	0.42	0.19 to 0.65
Swelling	0.40	0.23 to 0.58
Flaking	0.24	−0.006 to 0.48
Vesiculation	−0.006	−0.016 to 0.0042
Hyperpigmentation	−0.024	−0.043 to −0.0055
Hypopigmentation	−0.0074	−0.021 to 0.0062
Atrophy	−0.0059	−0.016 to 0.0042
Scarring	N/A^c^	N/A

a Interpretation of κ values: 0-0.20 (slight), 0.21-0.40 (fair), 0.41-0.60 (moderate), 0.61-0.80 (substantial), and 0.81-1.00 (almost perfect).

b Lesion resolution was graded at four levels (incomplete, <50%, >50%, and complete) and as a binary outcome (complete resolution vs incomplete resolution).

c N/A: not applicable.

**Table 3.** Interrater agreement for image-derived metrics of cryosurgery response by quality (n=195).

Image derived metric	Good-quality image (n=151)		Poor-quality image (n=44)	
	κ value^a^	95% CI	κ value	95% CI
Four point scale				
Lesion resolution^b^	0.75	0.66 to 0.84	0.50	0.278 to 0.73
Complete versus incomplete				
Lesion resolution	0.64	0.48 to 0.80	0.14	−0.23 to 0.52
Local skin response metrics				
Erythema	0.68	0.59 to 0.77	0.51	0.265 to 0.75
Crusting	0.50	0.30 to 0.70	0.60	0.29 to 0.91
Local skin response composite	0.70	0.62 to 0.79	0.62	0.402 to 0.85
Erosion	0.54	0.29 to 0.79	−0.019	−0.048 to 0.0155
Scaling	0.47	0.20 to 0.73	0.23	−0.11 to 0.57
Flaking	0.22	−0.039 to 0.48	0.31	−0.16 to 0.77
Swelling	0.42	0.211 to 0.62	0.36	0.029 to 0.68
Vesiculation	−0.0076	−0.021 to 0.0055	N/A^c^	N/A
Hyperpigmentation	−0.024	−0.047 to −0.0008	−0.031	−0.066 to 0.0040
Hypopigmentation	−0.0089	−0.025 to 0.0071	N/A	N/A
Scarring	N/A	N/A	N/A	N/A
Atrophy	−0.0067	−0.018 to 0.0045	N/A	N/A

a Interpretation of κ values: 0-0.20 (slight), 0.21-0.40 (fair), 0.41-0.60 (moderate), 0.61-0.80 (substantial), and 0.81-1.00 (almost perfect).

b Lesion resolution was graded at four levels (incomplete, <50%, >50%, and complete) and as a binary outcome (complete resolution vs incomplete resolution).

c N/A: not applicable.

### Predictors of Lesion Response and Patient-Rated Side Effects

**Figure 2.**
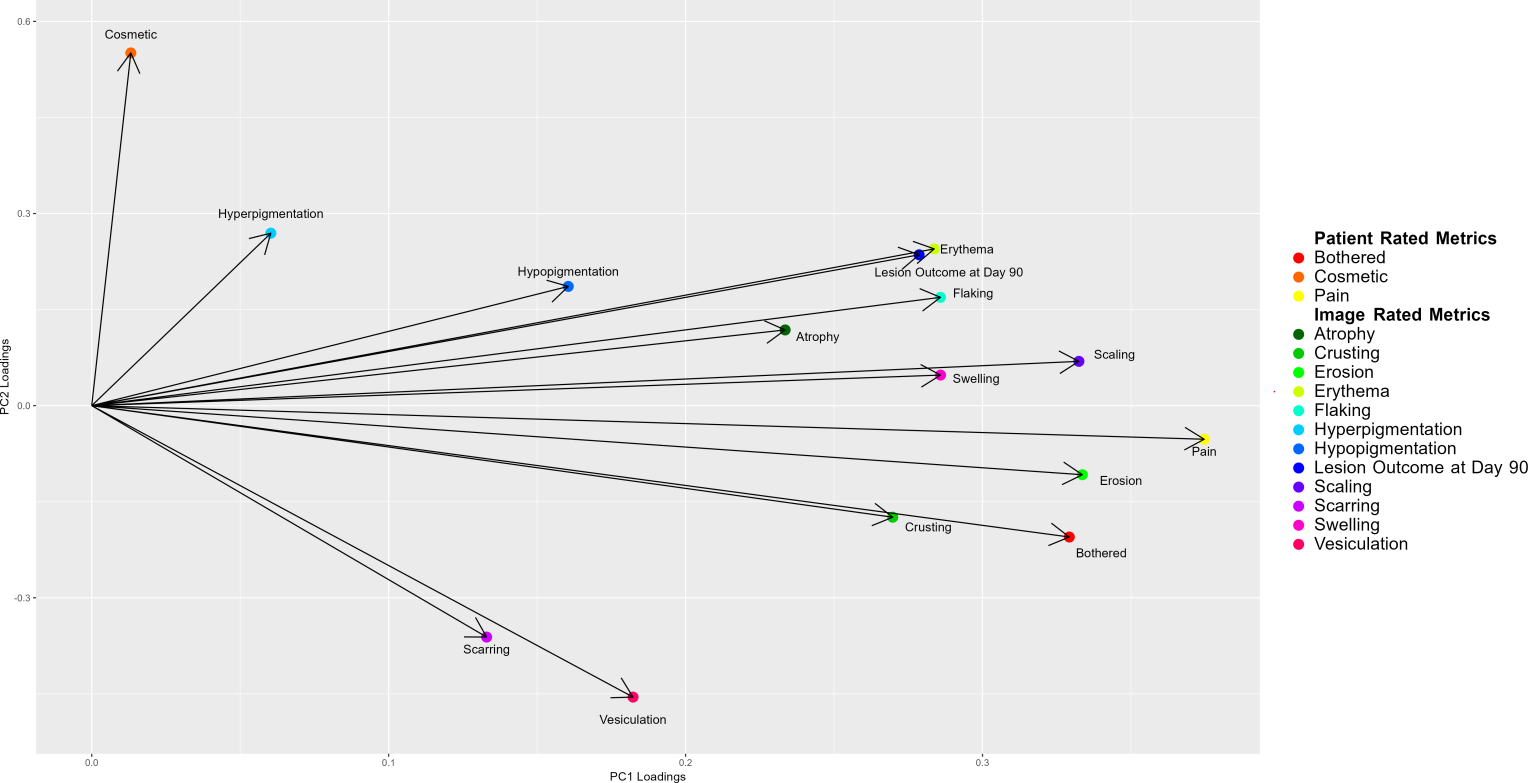
Biplot of principal component analysis loadings for peak image-rated outcomes and patient-reported adverse reactions. This biplot showcases the first two principal components for principal component analysis performed on the peak local skin response values and patient-reported outcomes with the 90-day lesion resolution (26 lesions). The vector direction indicates the pattern of variance, and the length denotes the strength of contribution to the principal component. The variable “Erythema” is closely clustered with the “Lesion Outcome at Day 90,” indicating its potential as a predictor of long-term resolution. Additionally, “Pain” is closely clustered with “Erosion,” and “Bothered” is closely clustered with “Crusting,” reflecting a shared variance among these adverse reactions. PC: principal component.

Peak responses for image-rated metrics were determined by averaging the highest grades assigned by rater 1 and rater 2 across all time points. Peak values for patient-rated adverse reactions were determined by identifying the maximum scores reported for cosmetic impact, level of bother, and pain across all time points. The peak LSR score had a moderate positive association with the lesion response at 90 days (Spearman ρ=0.556, *P*=.01), a moderate positive association with maximum pain (Spearman ρ=0.643, *P*<.001), and a moderate positive association with the maximum rated degree bothered (Spearman ρ=0.545, *P*=.002), all suggesting that lesions with a higher visually assessed LSR score are more likely to be painful, bothersome, and appear resolved long term. The cosmetic outcome at 90 days was also moderately associated with lesion outcome at 90 days (Spearman ρ=0.591, *P*=.008). Figure 2 shows principal component analysis of peak image-based metrics, peak patient-rated adverse reactions, and lesion resolution at day 90. A biplot of principal component 1 and principal component 2 loadings shows that the lesion outcome at day 90 is most closely clustered with peak erythema, suggesting that changes associated with increased local redness may be the most important factor for long-term lesion resolution. Pain is closely clustered with erosion, and being bothered is closely clustered with crusting.

## Discussion

### Principal Findings and Comparison With Prior Work

The findings of this study support the feasibility of using store-and-forward photos for remotely documenting lesion response to destructive therapy. In summary, we found that the image-derived response of keratoses to cryosurgery can be reliably labeled using photos submitted by patients, and that the LSR metrics correlate with patient-reported outcomes like pain and long-term lesion resolution. Several parameters, such as lesion response, the LSR index, and erythema, showed substantial agreement between raters. However, the agreement was minimal for vesiculation, hyperpigmentation, and hypopigmentation, although these were rare events and may have been underpowered to detect any significant agreement. When comparing the agreement of image-derived metrics by photo quality, a higher concordance was noted for lesion resolution and several other response indicators for high-quality photos. However, several response metrics did not vary by photo quality. This suggests that even with a standardized grading system, both rater judgment and photo quality may influence grading of lesion response.

Of the submitted images, 77% (151/195) were deemed adequate for assessment. This surpasses a recent study where 55.1% (1985/3600) of patients submitted photos of various rashes or lesions that were of sufficient quality for medical decision-making [8]. The design of the mobile app, which provided access to baseline images and quality reminders, along with explicit instructions, likely contributed to image quality. We previously reported on the usability of the app in the first 8 patients, who reported ease of use with a mean score of 4.4 out of 5 [9].

Cryosurgery treatment is unstandardized, and our results showed a wide range of apertures, spray times, spray distances, and cycles. This may lead to inadequate treatment for patients and/or bothersome adverse reactions. In our study, 58% of lesions were considered resolved by at least one rater, slightly below the reported rates of 63% to 88% [10-15]. Still, cryosurgery was well tolerated at day 90, although it is worth noting that a subset of patients still reported long-term pain. Reported tolerability of cryosurgery varies widely, with a recent meta-analysis documenting pain or burning in 7% to 22% of cases, crusting in 6%, and discoloration or scarring in 33% [10-16]. This shows there still may be a subset of patients who experience long-term sequelae and highlights the need for personalized treatment approaches to minimize adverse effects, although this study is not powered to evaluate those specifics.

The lesion outcome at 90 days was moderately associated with the peak LSR, highlighting that more robust inflammation may lead to greater long-term resolution. As the LSR appeared to peak between day 3 and day 7, early virtual follow-up may offer insights into the probability of long-term resolution. Still, principal component analysis suggests that lesion resolution may not be closely associated with peak patient-reported outcomes of pain, cosmesis, and degree bothered, highlighting the utility of image-based approaches to evaluate treatment efficacy. Future studies may aim to utilize machine learning approaches that combine reliable image-based metrics with patient-reported outcomes to predict treatment success [17].

### Limitations

The study faced limitations; most notably, there was a lack of diversity of skin type, which is important to study because both imaging and response to destructive therapy can vary based on skin type or tone. Future studies should aim to recruit patients that reflect the general population and include destructive therapies often used for patients of darker skin tones (eg, electrodessication). This study is also limited by its small sample size and the lack of in-person assessments. Specifically, a diagnosis of keratosis often relies on tactile cues that were not conducted during follow-up. Additionally, visual assessment of lesion resolution was sometimes difficult to distinguish from persistent posttreatment cosmetic adverse reactions (eg, erythema, crusting, and pigment alterations), which could have affected the grading of both lesion resolution and/or LSR. Lastly, the generalizability of the study is limited by the small number of raters and the single-center setting.

### Conclusions

Our research demonstrates that certain image-derived skin response metrics can be reliably labeled from patient-submitted photos and are associated with both lesion responses and some patient-reported outcomes. These findings emphasize the potential of teledermatology for assessing the response to destructive therapies and highlight the limitations of visual grading of the LSR from non-standardized photos. These findings pave the way for future studies aimed at integrating image-based and patient-rated metrics for outcome assessment.

## Supplementary material

10.2196/63467 Multimedia Appendix 1 Patient self-imaging protocol and survey instrument.

10.2196/63467 Multimedia Appendix 2 Image-based rater grading instructions and interface.

10.2196/63467 Multimedia Appendix 3 Frequency of image-derived metrics by rater agreement.

10.2196/63467 Multimedia Appendix 4 Patient-rated side effects over time.

## Abbreviations

LSR: local skin response
