# Supplementary material for: Remote Monitoring of Cryosurgery Response Using a Smartphone App: Prospective Study
Source: JMIR Dermatol. 2026 Mar 18;9:e63467. doi: 10.2196/63467 (PMC12998606; doi:10.2196/63467)
Supplement: Multimedia Appendix 1 [file derma-v9-e63467-s001.docx]

**Multimedia Appendix 1:** **Patient Self-Imaging Protocol and Survey Instrument**

Data Collection

The patient self-imaging app (Canfield Capture Mobile App, Canfield Scientific, Parsippany, NY) was downloaded onto the patient’s phone, and a close-up and overview photo were taken by the patient for up to 3 lesions. The selected lesions were treated with cryotherapy and distance between the nozzle and lesion, length of spray, and number of cycles were recorded.

Patients were provided with a reference guide for taking good clinical photographs at home (Figure S1). It instructed participants to take a global image of their lesion(s) at 30 cm and a close-up image of the lesion(s) at 15 cm using indoor, well-lit lighting with a parallel tilt. Participants were encouraged to resubmit if the photo was out of focus or not centered. For post treatment visit 0, patients selected the location of the lesion on a body map, took an overview photo and confirmed it met the quality check list: Only skin or solid color in the background, bright even lighting, no shadow or glare, lesion centered and in frame, lesion in focus, and phone directly above skin and zoomed in. If it did not meet the quality checklist they were asked to retake. They tagged the lesion in the overview photo and took a close-up photo confirming it met the quality check list. On post treatment days 3, 7, 10, 14, 30, 60, and 90, patients clicked on an existing overview photo of their lesion(s) and took a new “close up” and “overview” photo of their treated lesion(s), confirming each time it was consistent with the image quality checklist. They then answered a questionnaire (Table S1). Participants were asked three questions. “How would you describe the cosmetic outcome (appearance) of your treatment?” and rated it as poor, fair, good, very good, or excellent. Second, “How often are you bothered by the side effects of your treatment?” and rated it from 1 (never bothered) to 7 (always bothered). Third, “Rate your treatment related pain on the following scale” and rated it from 0 (no pain) to 10 (unimaginable). A free text box was available for additional comments. The post treatment Day 0 visit was completed while on site at clinic, while all other post-treatment visits were conducted at home. Email reminders were sent to participants the day before and day of intended entry and the day after if the survey was not completed.

**Figure S1.** Photo quality information sheet given to patients on Day 0


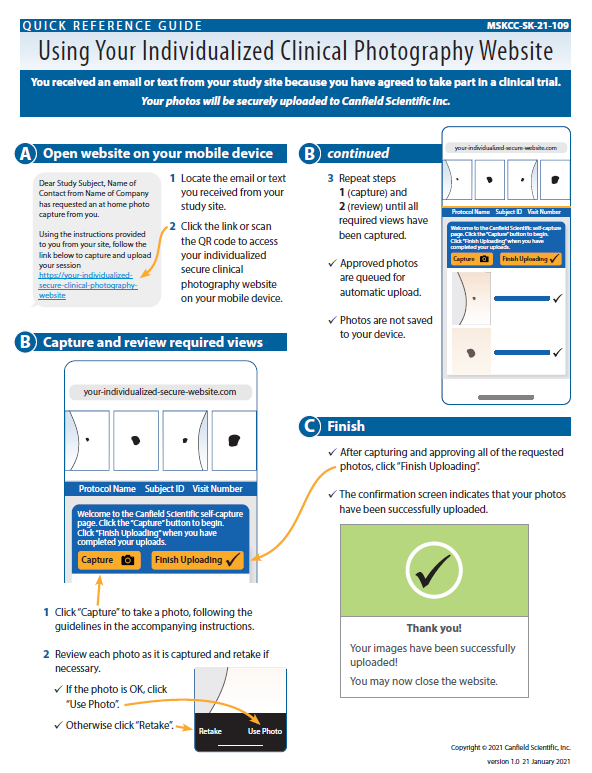


**Table S1.** Patient Questionnaire completed on Post-Treatment Day 0, 3, 7, 10, 14, 30, 60 and 90. Cosmetic Appearance was not completed on Day 0

| **Q1. How would you describe the cosmetic outcome (appearance) of you treatment?** | | | | | | | | | | | | | | |
| --- | --- | --- | --- | --- | --- | --- | --- | --- | --- | --- | --- | --- | --- | --- |
| Poor | | | Fair | | | Good | | | | Very Good | | | Excellent | |
| **Q2 How often are you bothered by the side effects of your treatment** | | | | | | | | | | | | | | |
| 1 (Never Bothered) | | 2 | | 3 | | | 4 | | 5 | | | 6 | 7 (Always Bothered) | |
| **Q3 Rate your treatment related pain on the following scale:** | | | | | | | | | | | | | | |
| 0 – No pain | 1 – Very mild | | | | 2 – Discomforting | | | 3 – Tolerable | | | 4 – Distressing | | | 5 – Very Distressing |
| 6 – Intense | | | 7 – Very Intense | | | 8 – Utterly horrible | | | | 9 – Excruciating, unbearable | | | 10 – Unimaginable, Unspeakable | |
| **If applicable, please describe any other side effects you are experiencing due to treatment (eg. Scarring, pigmentation changes, swelling, numbness, and so on)** | | | | | | | | | | | | | | |
| Free text box | | | | | | | | | | | | | | |
