# Supplementary material for: Remote Monitoring of Cryosurgery Response Using a Smartphone App: Prospective Study
Source: JMIR Dermatol. 2026 Mar 18;9:e63467. doi: 10.2196/63467 (PMC12998606; doi:10.2196/63467)
Supplement: Multimedia Appendix 2 [file derma-v9-e63467-s002.docx]

**Multimedia Appendix 2: Image-Based Rater Grading Instructions and Interface**

*Independently Rated Measurements*

On a PowerPoint interface, a pretreatment photo of the lesion at baseline, identified via an arrow, was compared to both a close-up photo and an overview photo of the lesion taken at one post-treatment timepoint (Figure S1). On the right side of the slide were questions assessing lesion resolution, local skin response, and photo quality. Using the grouping of pre-treatment and post-treatment photos, each post-treatment timepoint was independently rated for atrophy, crusting, erythema, flaking, scaling, swelling, erosion/ulceration, hyperpigmentation, hypopigmentation, scaring, and vesiculation from 0-4 based on an adapted previously established Local Skin Response (LSR) scale[8] (Table S1). Paired pre- and post-treatment images for each timepoint (day 0, 3, 7, 10, 14, 30, 60, 90) were presented to the reviewers randomly across patients and timepoints. Lesion resolution was visually rated as complete response or incomplete response. In lesions rated ‘incomplete’, response was graded as >50% resolved, <50% resolved, or no change. Local skin response index was calculated by combining erythema (0-4), crusting (0-4), swelling (0-4), vesiculation/pustulation (0-4), erosion/ulceration (0-4), and maximum score for flaking or scaling (0-4) for a composite score of 0-24. Photos were visually assessed for quality and rated as “Poor” (cannot identify lesion resolution or local skin response based on the photo) or “Good”. Two raters, dermatologists specialized in pigmented lesion diagnosis, each rated all of the 195 image pairs.

**Figure S1.** Example of PowerPoint grading interface


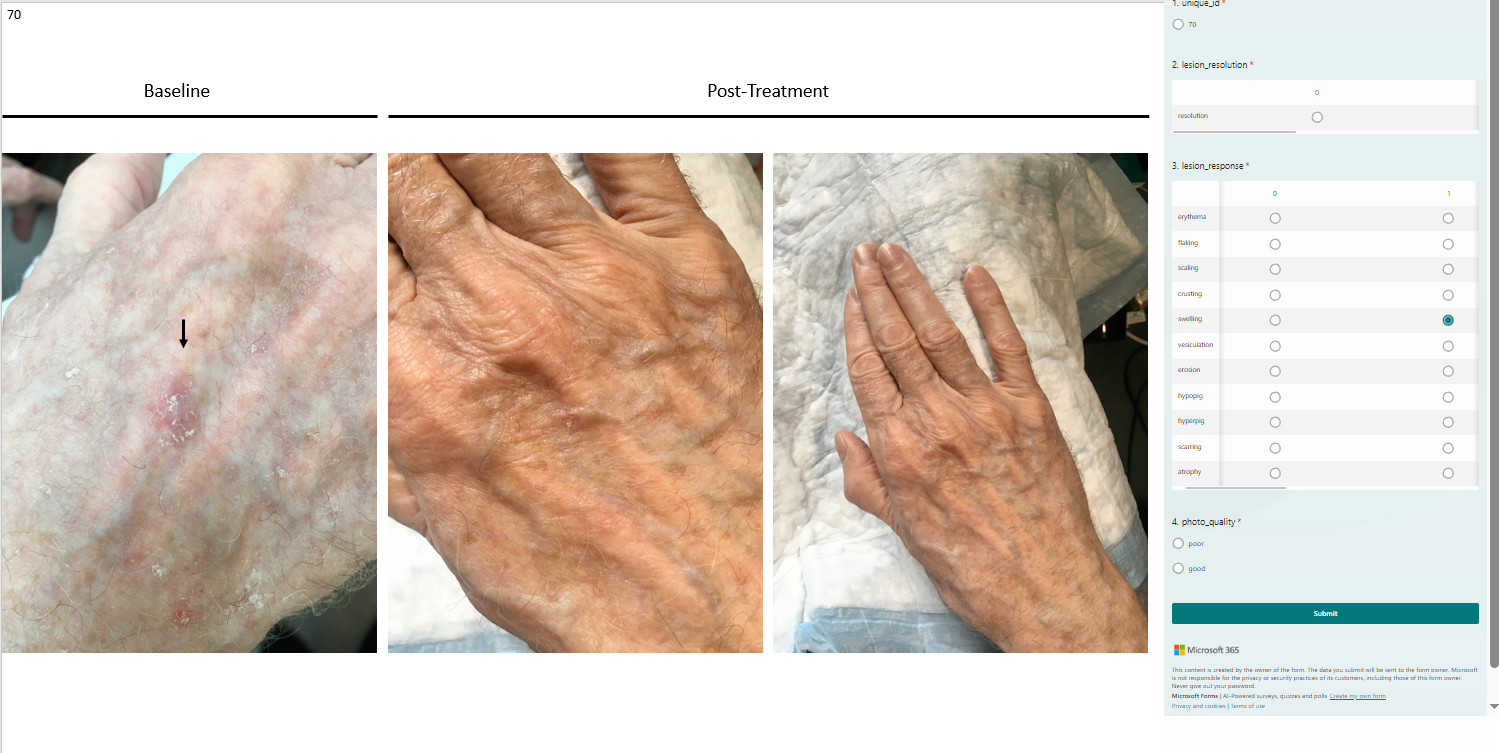


**Table S1.** Directions for Raters for Grading Image Based Metrics.

**Directions:** Please make all assessments of post-treatment lesions (closeup and overview) comparison to pre-treatment baseline. If an outcome measure is equivalent to baseline, please select 0.

| **Objective** |  |  |  |  |  |
| --- | --- | --- | --- | --- | --- |
| Lesion Resolution **Compared to baseline** | No change (0) | Incomplete (1) | Incomplete (2) | Complete (3) |  |
|  |  | <50% | >50% |  |  |
| Erythema | None or equivalent/less than baseline (0) | Slightly pink <50% (1) | Pink or light red >50% (2) | Red, restricted to treatment area/lesion area (3) | Red extending out of lesion area (4) |
| Flaking/scale | Not present or equivalent/less than baseline (0) | Isolated scale, specific to lesion (1) | Scale <50% (2) | Scale >50% (3) | Scale extending outside treatment area (4) |
| Crusting | None or equivalent/less than baseline (0) | Isolated crusting (1) | Crusting <50% (2) | Crusting >50% (3) | Crusting outside treatment area (4) |
| Swelling | Not present (0) | Slight (1) | Swelling confined to lesion (2) | Visible swelling slightly beyond lesion (3) | Marked swelling outside treatment area (4) |
| Vesiculation/Pustulation | None (0) | Vesicles only (1) | Transudate or pustules with/without vesicles <50% (2) | Transudate or pustules with/without vesicles >50% (3) | Transudate or pustules, with or without vesicles extending outside treatment area (4) |
| Erosion/Ulceration | None | Lesion specific erosion (1) | Erosion extending beyond individual lesions (2) | Erosion >50% (3) | Black eschar or ulceration (4) |
| Hyperpigmentation | None or equivalent to baseline (0) | Isolated (1) | <50% of lesion (2) | >50% of lesion (3) | Extending beyond lesion area (4) |
| Hypopigmentation | None or equivalent to baseline (0) | Isolated (1) | <50% of lesion (2) | >50% of lesion (3) | Extending beyond lesion area (4) |
| Scaring | None or equivalent to baseline (0) | Isolated (1) | <50% of lesion (2) | >50% of lesion (3) | Extending beyond lesion area (4) |
| Atrophy | None or equivalent to baseline (0) | Isolated (1) | <50% of lesion (2) | >50% of lesion (3) | Extending beyond lesion area (4) |
| Photo Quality | Poor Quality | Good quality |  |  |  |
