# Supplementary material for: Remote Monitoring of Cryosurgery Response Using a Smartphone App: Prospective Study
Source: JMIR Dermatol. 2026 Mar 18;9:e63467. doi: 10.2196/63467 (PMC12998606; doi:10.2196/63467)
Supplement: Multimedia Appendix 3 [file derma-v9-e63467-s003.docx]

**Multimedia Appendix 3: Frequency of Image-Derived Metrics by Rater Agreement**

**Table S1:** Frequency of Image Reported Metrics Across All Timepoints by Rater (N=29 Total)

| **Metric** | **No Raters** | **One Rater** | **Both Raters** |
| --- | --- | --- | --- |
| **Erythema** | 2 | 27 | 26 |
| **Flaking** | 10 | 19 | 13 |
| **Scaling** | 9 | 20 | 15 |
| **Crusting** | 8 | 21 | 15 |
| **Swelling** | 5 | 24 | 18 |
| **Vesiculation** | 26 | 3 | 0 |
| **Erosion** | 14 | 15 | 9 |
| **Hyperpigmentation** | 21 | 8 | 0 |
| **Scarring** | 28 | 1 | 0 |
| **Atrophy** | 26 | 3 | 0 |
| **Hypopigmentation** | 23 | 6 | 1 |
