# Supplementary material for: Remote Monitoring of Cryosurgery Response Using a Smartphone App: Prospective Study
Source: JMIR Dermatol. 2026 Mar 18;9:e63467. doi: 10.2196/63467 (PMC12998606; doi:10.2196/63467)
Supplement: Multimedia Appendix 4 [file derma-v9-e63467-s004.docx]

**Multimedia Appendix 4: Patient-Rated Side Effects Over Time**


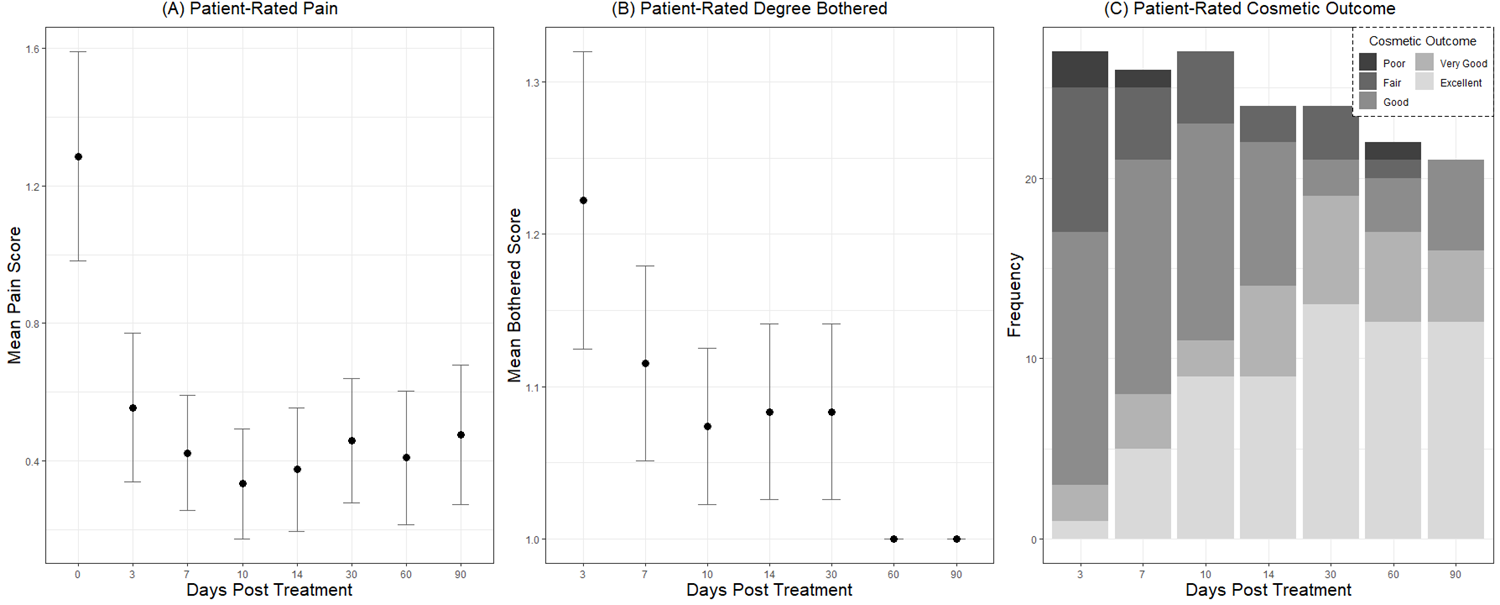


***Figure S1:*** ***Patient Rated Side Effects following Cryotherapy.*** (A) Mean reported pain following cryotherapy over time. Patients rated pain from 0 (no pain) to 10(unimaginable pain). While mean pain decreased after day 0, there was still a subset of patients who had persistent pain at day 90. (B) Mean reported degree bothered following cryotherapy over time. Patients rated degree bothered from 1 (never bothered) to 7(always bothered). No patients were bothered at day 60 or 90. (C) Frequency and distribution cosmetic outcome rating following cryotherapy. Ratings ranged from ”poor” to ”excellent.” At day 90, 100% of patients rated cosmetic outcome as good, very good, or excellent.
